# A186 CHANGES IN THE CLINICAL PHENOTYPE AND BEHAVIOR OF PEDIATRIC LUMINAL CROHN’S DISEASE AT DIAGNOSIS IN THE LAST DECADE

**DOI:** 10.1093/jcag/gwab049.185

**Published:** 2022-02-21

**Authors:** S Sassine, M Savoie-Robichaud, Y Lin, L Djani, C Cambron-Asselin, M Qaddouri, S Fadela Zekhnine, K Grzywacz, V Groleau, M Dirks, É Drouin, U Halac, V Marchand, C Girard, O Courbette, N Patey, D Dal Soglio, C Deslandres, P Jantchou

**Affiliations:** Centre Hospitalier Universitaire Sainte-Justine, Montreal, QC, Canada

## Abstract

**Background:**

Crohn’s disease (CD) triggers are incompletely understood and the incidence of the disease has been increasing.

**Aims:**

The aims of this study were to describe the trends in the clinical, endoscopic, histological, and laboratory characteristics of pediatric CD during the last decade and to describe the seasonal variation of disease presentation at diagnosis.

**Methods:**

Patients under 18 years old and diagnosed between 2009 and 2019 were included. Patients clinical, endoscopic, histological, and laboratory data were collected from the medical records. Data were analyzed for the cohort as a whole and according to diagnostic periods (2009–2014 and 2015–2019) and seasons.

**Results:**

654 patients were included in the study. The total number of incident CD cases significantly increased yearly. Patients diagnosed between 2015 and 2019 were younger at diagnosis (OR: 2.30, p<0.0001), had more perianal diseases (OR= 2.30, p<0.001) and more intestinal biopsy granulomas (OR= 1.61, p=0.003) as compared to the 2009–2014 cohort. Also, there was a strong association between intestinal biopsy granulomas, young age at diagnosis and perianal fistulas or abscesses; the presence of granulomas was associated with greater perianal involvements (OR= 2.25, p<0.001) and younger age at diagnosis (OR = 0.90, p=0.0002). PCDAI and SES-CD scores at diagnosis, disease location and behavior and laboratory markers did not change over time.

There were fewer CD diagnosis during winter. The highest vitamin D levels in patients occurred in summer and fall, but the majority of patients had, regardless of the season of diagnosis, severe vitamin D deficiency (the median vitamin D level was 60.0 nmol/L in summer and fall compared to 47.0 nmol/L in winter-spring, p=0.003). Vitamin D levels at diagnosis are inversely correlated with PCDAI (Pearson correlation coefficient = -0.19, p=0.03) and SES-CD (-0.20, p=0.04). Patients diagnosed in fall had lower PCDAI and SES-CD scores, less failure to thrive, less digestive symptoms and less extensive digestive involvement. Colonic disease was significantly more frequent during summer and fall (27.3% of patients diagnosed in summer and fall versus 18.2% of cases in winter and spring, p=0.01).

**Conclusions:**

The disease phenotype has changed over the years and there are important seasonal trends in the frequency and severety of the disease suggesting possible disease triggers. Our findings provide interesting avenues for future research, such as identifying the clinical significance of granulomas, vitamin D deficiency and microbiota on pediatric CD activity.

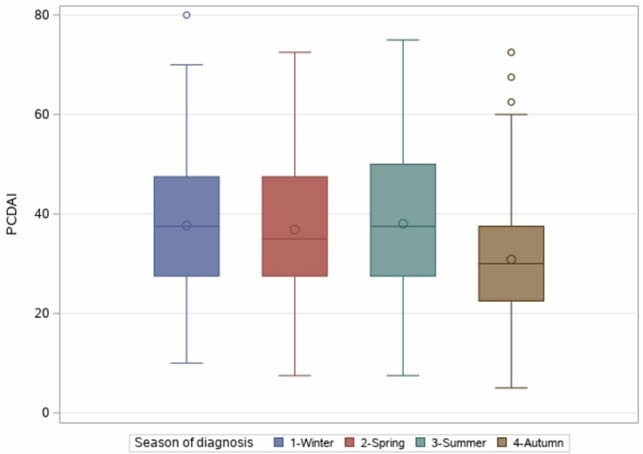

PCDAI at diagnosis according to the season.

**Funding Agencies:**

NoneFonds Recherche Santé Québec / Fondation du CHU Sainte-Justine

